# *In Vitro* Antioxidant Activity of Selected 4-Hydroxy-chromene-2-one Derivatives—SAR, QSAR and DFT Studies

**DOI:** 10.3390/ijms12052822

**Published:** 2011-04-29

**Authors:** Milan Mladenović, Mirjana Mihailović, Desanka Bogojević, Sanja Matić, Neda Nićiforović, Vladimir Mihailović, Nenad Vuković, Slobodan Sukdolak, Slavica Solujić

**Affiliations:** 1 Department of Chemistry, Faculty of Science, P.O. Box 60, University of Kragujevac, Radoja Domanovića 12, 34000 Kragujevac, Serbia; E-Mails: nneda@kg.ac.rs (N.N.); vladam@kg.ac.rs (V.M.); nvukovic@kg.ac.rs (N.V.); duda@kg.ac.rs (S.S.); ssolujic@kg.ac.rs (S.S.); 2 Department of Molecular Biology, Institute for Biological Research, University of Belgrade, Bulevar Despota Stefana 142, 11000 Beograd, Serbia; E-Mails: mista@ibiss.bg.ac.rs (M.M.); dekana@ibiss.kg.ac.rs (D.B.); 3 Department of Biology end Ecology, Faculty of Science, P.O. Box 60, University of Kragujevac, Radoja Domanovića 12, 34000 Kragujevac, Serbia; E-Mail: msmaticsanja@yahoo.com

**Keywords:** 4-hydroxycoumarins, antioxidant activity *in vitro*, DFT, BDEs, QSAR, design

## Abstract

The series of fifteen synthesized 4-hydroxycoumarin derivatives was subjected to antioxidant activity evaluation *in vitro*, through total antioxidant capacity, 1,1-diphenyl-2-picryl-hydrazyl (DPPH), hydroxyl radical, lipid peroxide scavenging and chelating activity. The highest activity was detected during the radicals scavenging, with **2b**, **6b**, **2c**, and **4c** noticed as the most active. The antioxidant activity was further quantified by the quantitative structure-activity relationships (QSAR) studies. For this purpose, the structures were optimized using Paramethric Method 6 (PM6) semi-empirical and Density Functional Theory (DFT) B3LYP methods. Bond dissociation enthalpies of coumarin 4-OH, Natural Bond Orbital (NBO) gained hybridization of the oxygen, acidity of the hydrogen atom and various molecular descriptors obtained, were correlated with biological activity, after which we designed 20 new antioxidant structures, using the most favorable structural motifs, with much improved predicted activity *in vitro*.

## 1. Introduction

Overall cell health depends on the balance between formation and elimination of free radicals [1]. Free radicals, which originate both in normal or pathological metabolic transformations, host-defense against undesirable invasion (chemical or biological), or host-response to a disturbance of the tissues’ integrity (due to trauma, cellular damage, *etc*.), may begin chain reactions initiated mostly by reactive oxygen species (ROS) [1]. They are, normally, continuously generated in the living cell, in low amounts by the transfer of one electron to an oxygen molecule during respiration chain and cellular immunization reactions [2] and are therefore needed for the normal redox-signaling and self-defense of the host [1]. Still, superoxide anion (O_2_^−^) and hydroxyl radical (OH), in increased concentrations, can induce oxidative stress and cellular damage by altering the biological activities of lipids, proteins, DNA and carbohydrates [3], even to cellular death [1]. ROS are associated with incidence of heart diseases, thrombosis [4], hypertension [5], Alzheimer’s and Parkinson’s diseases [6] and cancer over the radical induced DNA double-strain breaks [7].

Some substances, like hydroxyl-coumarins [8], directly recombine free radicals and interrupt the initiation and/or propagation of the induced chain reactions. Due to the typical phenolic behavior [1] they act as potent metal chelators and free radical scavengers, resulting in a powerful antioxidant effect. Their antioxidant behavior could be applied in fat and oily foods to prevent oxidative deterioration, to replace known synthetic antioxidants. Deterioration of food quality occurs during processing and storage, and is related to presence of oxygen and oxidative processes [9]. To show antioxidant activity, a coumarin derivative has to possess at least one hydroxy1 group [10].

Due to their great importance, the structural aspects of both natural and synthetic coumarins as antioxidant compounds have been evaluated using the structure-activity relationships (SAR) and quantitative structure-activity relationships (QSAR) methodology.

Thus, the evaluation of coumarin isolates from *Cortex Fraxini* [11], *Geranium wallichianum* [12] and Korean medicinal plants [13] highlights the presence of catechol moiety and oxygen containing scaffold in C-6 and C-7 positions of the coumarin core for antioxidant activity. Also, the α-pyrone coumarin ring increases free radical scavenging activity, antilipid peroxidation ability and has a suppressive effect on enzymes [13]. Synthetic compounds, 4-methylcoumarins [14], substituted 7- or 8-hydroxybenzo[f]-coumarins, 6-hydroxybenzo[h]-coumarins and 7-azomethinecoumarins were tested for their antioxidant ability *in vitro* [15] and for their ability to interact with 1,1-diphenyl-2-picryl-hydrazyl (DPPH) stable free radical, scavenging of the superoxide anion and inhibition of lipid peroxidation, too [16].

Thereafter, this paper presents the antioxidant potential *in vitro* of various C-3 carbonyl/carboxyl (**2**–**8b**) [17] and *N*-thiazole (**2**–**10c**) [18] 4-hydroxy-2*H*-chromen-2-one derivatives introducing the 4-hydroxy group as highly potent in reducing chain reaction processes and coumarin C-3 scaffolds as good hydroxyl radical scavengers and metal chelators. The structure potential of our derivatives was confirmed by SAR, QSAR and Density Functional Theory (DFT) studies, which were used for the successful design of 20 novel improved coumarin antioxidant structures.

## 2. Results and Discussion

### 2.1. Determination of Total Antioxidant Capacity

Results of the total antioxidant capacity are expressed as μg equivalents of ascorbic acid per milliliter (Table 1), demonstrating level of coumarin activity as TAC = 26.76 – 742.67 μg/mL. The derivation of the compound **1** at C-3 position resulted in the increase of the total antioxidant potential within both tested groups.

The examined compounds **2**–**8b** contain carboxyl or ester group *trans-*oriented towards the methyl group of the prop-1-en scaffold. The most active compound was **7b**, a (*E*)-2-cyano-3-but-2-enoic acid derivative (TAC = 324.01 μg/mL). After the substitution of cyano group in **7b** by carboxyethyl (**2b**; 278.24 μg/mL) and two acetyl (**6b**; 212.12 μg/mL) groups, activity was diminished. The decreasing order in the activity was **7b** > **2b** > **6b** > **4b** > **1** > **8b** > **3b**. Among the *N*-thiazole derivatives **2**–**10c**, with the **4c** > **2c** > **7c** > **8c** > **6c** > **3c** > **9c** > **10c** decreasing activity, the *N*-thiazole *p*-sulfonic acid derivative, **4c**, was pointed out as a very promising one, with the highest TAC value of 742.67 μg/mL. The *O*-hydroxybenzoic acid (**2c**), tolyl (**5c**, **8c**) and *N*,*N*-diethyl (**7c**) derivatives also presented notable potential, in the range of 198.84 μg/mL to 514.24 μg/mL.

In accordance with the results presented for the other activities (Sections 2.2–2.5), the total antioxidant capacity is also presented as TAC_50_ values (Table 1) which are used in the QSAR study.

Since the total antioxidant capacity assay describes the possibility of the tested compound to reduce Mo(VI) to Mo(V), we considered HOMO-LUMO gap as a way to explain coumarin oxidation ability. The smaller the gap is, the more easily molecules will be excited [19], *i.e.*, the test compound have higher potential to reduce Mo(VI). The range of Δ*E* HOMO-LUMO (Please see section 2.3.) was −8.53 eV (**4b**) to −7.05 eV (**2c**), suggesting potentially good activity. The high total antioxidant capacity had been obtained by compounds **7b** (Δ*E* HOMO–LUMO = −8.35 eV), **2c** (−7.05 eV) and **4c** (−7.77 eV), presenting fine correlation between the activity and the phisico-chemical characterization (Equation 3) of the compounds.

### 2.2. DPPH Radical Scavenging Activity

The *in vitro* activity of five coumarin derivatives **2b**, **6b**, **2c**, **4c** and **9c** (Table 1) was comparable with the standard values of ascorbic acid (**Asc**) and butylated hydroxytoluene (**BHT**) (30 minutes: ascorbic acid IC_50_ = 24.17 μg/mL, **BHT** IC_50_ = 8.62 μg/mL; 60 minutes: ascorbic acid IC_50_ = 15.61 μg/mL, BHT IC_50_ = 6.05 μg/mL).

After the 30 min period, compounds **2**–**8b** exhibited the following antioxidant potential: **6b** > **2b** > **8b** > **4b** > **3b** > **1** > **7b**, outlining acetyl groups of the prop-1-en moiety in **6b** (IC_50_ equal to 5.14 μg/mL) as the most structurally favorable residues. The double carboxyethyl substitution of the prop-1-en moiety (**2b**) decreased the scavenging potential in a small manner (IC_50_ = 6.2 μg/mL). Still notable, but significantly lower activity (<50 μg/mL) was noticed for **3b**, **4b** and **8b** containing carboxymethyl or carboxyl *trans*-substituents, with various *cis*-oriented scaffolds. It is worth mentioning that **4b** was the only compound with *cis*-hydrogen. The presence of acetyl group (**1**) instead of prop-1-en scaffold has significantly reduced the activity. The lowest activity of the **7b** can be attributed to the influence of *cis*-cyano group. The prolongation of the test time has magnified the potential of **2**–**8b**, particularly within the triad **3b**, **4b** and **8b**, which presented excellent activity (IC_50_ = 8.8 − 11.28 μg/mL). The higher scavenging potential of **3b** with respect to **4b** was caused by *cis*-acetyl **3b** pharmacaphore. Once again, **6b** was the most potent scavenger.

**Figure 1.**
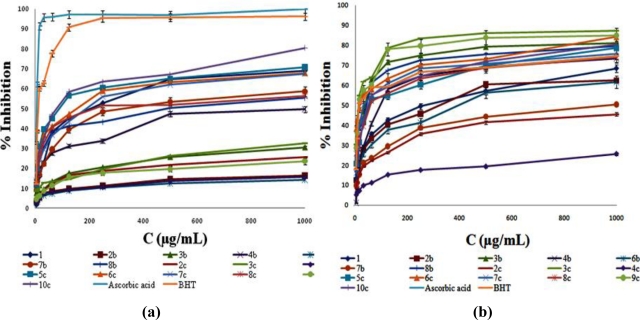
**(a)** DPPH radical scavenging activity in the 60th minute; and **(b)** determination of hydroxyl radical scavenging activity, *in vitro*.

Within the second test participants, **2**–**10c**, the strong hydrogen donor ability has distinguished the **4c** as the most powerful DPPH radical scavenger, regarding both test periods (IC_50_ values of 4.72 μg/mL and 3.54 μg/mL). The activity decreased in the raw **4c** > **2c** > **9c** > **3c** > **5c** (Figure 1, **a**). The *N*-thiazole motif linked with favorable *p*-SO_3_H group (**4c**) as well as the presence of the additional OH group (**2c**, IC_50_ equal to 4.9 μg/mL and 6.97 μg/mL) increased the antiradical activity. The high 30 min activity of the **2c** was not a surprise due to more look-a-like phenolic structure of the compound, related to increased number of the hydroxyl groups. The *m*-NO_2_ *N*-thiazole derivative (**9c**), reached full scavenging potential after 60 minutes test (IC_50_ = 4.79 μg/mL) thus overpowering the decreasing activity of **2c** in the same period. Despite the favorable structure, **2c** was the only compound that presented weaker potential after 60 min testing. The thiazole derivatives containing only *N*-tolyl (**5c** and **8c**), *N*,*N*-diethyl (**7c**) and *N*-naphtyl residues, presented a total lack of activity.

The scavenging potential of our compounds was: **4c** > **2c** > **6b** > **2b** > **BHT** > **9c** > **Asc** after 30 min, while the prolonged reaction time changed the activity in the following manner: **6b** > **4c** > **2b** > **9c** > **BHT** > **2c** > **3c** > **Asc**.

### 2.3. Inhibition of Lipid Peroxidation in Linoleic Acid Emulsion

During the four days long inhibition of lipid peroxidation by coumarin derivatives, the absorbance of the control sample at 500 nm has been increasing up to the maximal value in 72nd hour, and then, on the 96th hour the absorbance decreased due to the decomposition of linoleic acid hydroperoxides generated during the peroxidatition [20]. Consequently, we present the results of coumarin induced inhibition of lipid peroxidation until the 72nd hour (Table 2). **BHT** was excellent standard for this measurement with the I_50_ = 7.81 μg/mL.

During the observed 24–72 h time interval, a group of five tested compounds, **2b**, **6b**, **2c**, **4c** and **9c**, has presented significant lipid peroxide scavenging capacity, compared to **1** and **BHT**. Regarding the results, some patterns in the activity among derivatives could be obtained. Thus, the compounds **6b** (I_50_ = < 3.90 μg/mL; 10.13 μg/mL; 10.76 μg/mL for 24 h, 48 h and 72 h, respectively) and **9c** (I_50_ = 5.88 μg/mL; 10.06 μg/mL; 12.23 μg/mL) retained the level of activity after the second and third day. Compared to **BHT**, **6b** expressed 50% higher potential in the first 24 hours. Within the following group, **2c**, **4c** and **2b**, a small decrease in the activity on the 48th hour has been noticed, accompanied with amelioration on the third day of the test. The most active compound within the triad was **4c** (I_50_ < 3.90 μg/mL; 26.31 μg/mL; 10.09 μg/mL). Furthermore, a group of structurally similar compounds, **3b**, **4b** and **8b**, showed slightly lower radical scavenging intention than previously mentioned derivatives with I_50_ from 11.49 to 18.96 μg/mL on the 24th hour, 18.07 to 37.89 μg/mL on the 48th hour and from 10.46 μg/mL to 80.95 μg/mL on the final hour. The structural change of *cis*-hydrogen atom (**4b**) with acetyl (**3b**) or carboxymethyl (**8b**) group did not contribute to the activity.

The antioxidant capacity of an organic compound, whether natural or synthetic, is expressed as the ability of the compound to release hydrogen atom, *i.e.*, to undergo keto-enol tautomerisation [21]. The molecular bonds with a length between a single and double bonds, also increases the radical scavenging potential. Furthermore, low Bond Dissociation Enthalpies (BDE) values are often attributed to the high antioxidant potential [21,22].

**Figure 2.**
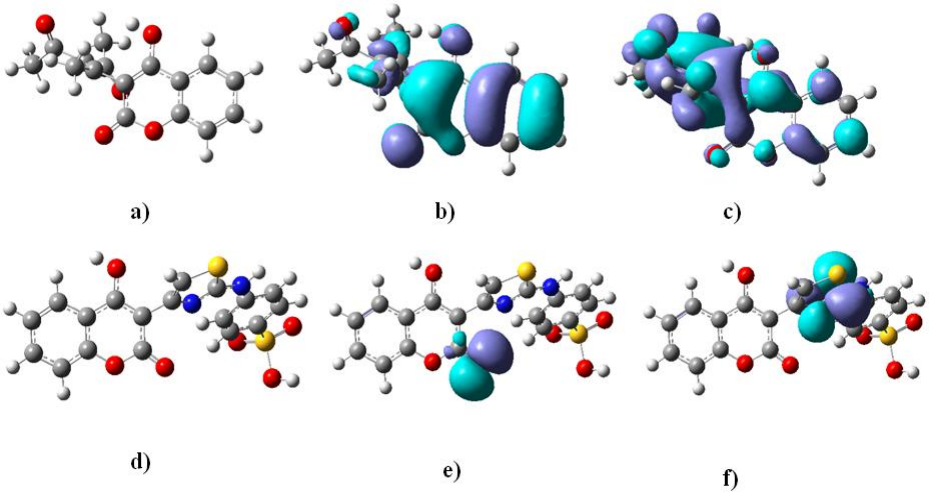
**(a)** and **(d)** 4-OH bonds of **6b** and **4c** with **(b)** and **(f)** HOMO; **(c)** and **(f)** LUMO orbitals of **6b** and **4c**, respectively.

The proton transfer from the antioxidant towards radical is common for both DPPH and lipid peroxide scavenging. Therefore, we present the DFT study on the coumarin 4-OH group which is responsible for antioxidant activity. As has been obtained by the single-point DFT calculations and presented in Figure 2**a** and **d**, the 4-hydroxyl group of the tested derivatives is highly enolized and the hydrogen atom is almost released towards available free radical. In the methanol solution, the coumarin structure is transformed into hinon-like (Figure 2**a** and **d**) and the 4-OH hydrogen atom is attached to oxygen by weak electrostatic interactions. Therefore, the hydrogen is suitable for abstraction by the free radical. Moreover, by NBO analysis, we concluded that the acidity of the 4-OH hydrogen is very significant for the activity. Although the hybridization of the oxygen atom in the bond is *sp^2^* by equations 0.7814(sp^1.67^)_O_ + 0.3926(s^100^)_H_ (**6b**) and 0.8947(sp^1.62^)_O_ + 0.4837(s^100^)_H_ (**4c**), it is still close to the *sp* one, regarding the (sp^1.50^) as the top border for *sp* hybridization, providing the relatively high acidity to the hydrogen atom. Furthermore, bond lengths of all our compounds show that the majority of the bonds are longer than double bonds and shorter than single bonds, indicating an extended conjugation with antioxidant properties [21]. The lengths of 4-OH bond in **6b** (*d* = 1.03 Å) and **4c** (*d* = 1.63 Å) are the longest ones (Table 3, D4), causing the low O-H bond dissociation enthalpy (Table 3, E1) [21,22], consequently facilitating the release of the proton towards radical. This influence of the bond length is also described by Equations (2) and (3). Beside the fact that the BDEs of **6b** (205.84 kcal/mol) and **4c** (239.26 kcal/mol) are higher compared to coumarin standards [22], the most decisive factor for the strong antioxidant activity is the hinon-like structure of coumarin compounds in the methanol solution and the great availability of the hydrogen. In agreement with these facts, the compounds **6b** and **4c** have shown excellent DPPH scavenging and lipid peroxidation activity. Compound **2c** also presented notable potential due to long the 4-OH bond length (*d* = 1.55 Å) and considerable acidity of the hydrogen atom: 0.6826(sp^1.73^)_O_ + 0.3726(s^100^)_H_. Still, this is the only compound possessing more than one OH group which possibly contributes to the activity.

### 2.4. Determination of Hydroxyl Radical Scavenging Activity

The hydroxyl radical scavenging activity of the **BHT** (OH_50_ equal to 33.92 μg/mL) has been impaired by ten compounds in the following order **4c** > **2c** > **7b** > **6b** > **2b** > **1** > **3c** > **9c** > **6c** > **3b** > **BHT**, with 1.5–82.5% higher potential (Table 2) (Figure 1**b**). All of the tested compounds were better hydroxyl radical scavengers than ascorbic acid. The activities of **4c** (OH_50_ = 5.94 μg/mL), **2c** (OH_50_ = 9.81 μg/mL), **7b** (OH_50_ = 9.89 μg/mL) and **6b** (OH_50_ = 14.32 μg/mL) are worthy of note. Generally, the influence of the *N*-thiazole group linked to coumarin core, along with the functional groups that extended the conjugation (*p*-SO_3_H, *p*-, *m*-NO_2_ and Ph-*o*-OH-*p*-COOH), contributed to higher activity of **2**–**10c** in comparison to **1**–**8b**. On the other hand, the presence of *cis*-cyano group in **7b** enhanced the hydroxyl radical scavenging activity for 40–89%, compared with **1**, **2b** and **6b**. The hydroxyl radical scavenging was the only test during which **1** showed a certain potential. The OH radical abstraction is favored due to extended conjugation in our compounds (Please see section 3.1.).

All presented data are in strong correlation with observed radical scavenging activity.

The abstraction of the OH radical occurs in LUMO orbitals of the coumarin double bond system (Figure 2, **c** and **f**) which is confirmed by Equation (4). The LUMO orbitals of **6b** (widespread trough molecule) and **4c** (thiazole N = C) are presented as the OH radical abstraction centers.

### 2.5. Measurement of Ferrous Ion Chelating Ability

Starting with *N*-thiazole *p*-NO_2_ derivative **3c**, (CE_50_ = 5.5 μg/mL), followed by *m*-NO_2_ derivative **9c** (CE_50_ = 7.76 μg/mL), all tested compounds, except **1**, had surpassed ferrous ion chelating ability (Table 2) of ascorbic acid (CE_50_ 76.31 μg/mL) and **BHT** (CE_50_ 85.48 μg/mL). The chelating effect in the first group of the compounds descended in following order **6b** > **2b** > **8b** > **3b** > **7b** > **4b** > **1**. The decrease in number of oxygen atoms, starting with **3b**, significantly reduced the chelating power. The influence of the carbonyl/carboxyl and *N*-thiazole residues on chelating activity was comparable.

The lactone part of coumarin derivatives can bind the iron atom, but that was not confirmed by Equation (5). Governed by the fact that the oxygen groups are crucial for the chelating ability [23], high activity of **6b**, **2b** and **8b** is assigned to the presence of numerous oxygen atoms), Equation (5). Interestingly, **2b** and **8b**, containing carboxyl groups, had lower activity than **6b** (two acetyl groups). The potential of the **3c** and **9c** depends on the binding of iron with nitro oxygen atoms.

### 2.6. QSAR Studies of the Antioxidant Activity

**Figure 3.**
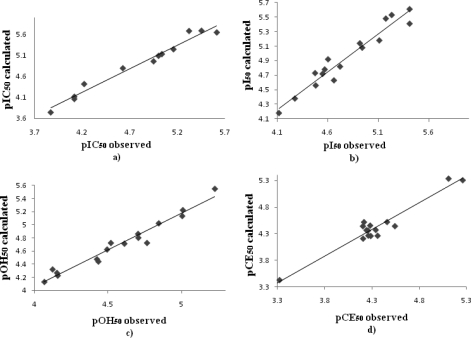
Plot of observed *vs.* calculated biological values of the training set compounds obtained from **(a)** Equation (2); **(b)** Equation (3); **(c)** Equation (4); **(d)** Equation (5).

QSAR studies were administrated to the antioxidant activity as a tool to describe and explain the activity of the tested coumarin derivatives. The results of the *in vitro* testing and the concentrations of the test compounds that reduce 50% of the initial free radical concentration were used for the QSAR study. Logarithmic values of the biological activity were used for the generation of the QSAR equations with relevant molecular descriptors (Figure 3). From the equations, we ascertained the possible mechanisms by which our coumarins occur as antioxidants.

#### 

##### QSAR study on total antioxidant capacity

(1) $$\begin{array}{c} {\text{pTAC}}_{50}=-8.46(\pm 0.32)+7.32(\pm 0.99)\;\text{HOMO}-2.69(\pm 0.47)\;\text{LUMO}\\ -9.46(\pm 1.48)\;Q_{0\text{H}}+0.6(\pm 0.1)\;Q_{\text{N}}-2.9(\pm 0.4)Q_{\text{S}}\\ \text{n}=15;\;\text{r}=0.992;\;\text{s}=0.032;\;\text{F}=37.426;{\text{Q}}^{2}=0.993\;\text{s-PRESS}\;=\;0.004 \end{array}$$

According to the model that describes total antioxidant capacity, Equation (1), the potential of the compounds is highly dependent of HOMO electron energies and the partial atomic charge of the thiazole ring nitrogen. The equation describes the thiazole center as electrone rich and as participant in the Mo(VI) reduction, as well as the HOMO orbitals of the compounds. The HOMO orbital of **4c** is the orbital of the coumarin lactone carbonyl double bond system (Figure 2), highlighting the importance of the coumarin residue in the mechanism.

##### QSAR study on in vitro 60 min DPPH scavenging activity

(2) $$\begin{array}{c} {\text{pIC}}_{50}=+6.72(\pm 0.48)-1.23(\pm 0.57)\;{\text{Q}}_{0\text{H}}+4.42(\pm 0.35)\;4\text{-OH}\;\text{bond}\;\text{length}\;+\;3.4(\pm 0.94)\\ Q_{\text{N}}-7.6(\pm 0.26)\;Q_{\text{S}} \end{array}$$

The high basicity of the thiazole nitrogen atom attenuates the strength of the 4-OH group C-O bond, making the hydrogen atom more acidic and more easily relaxed (Figure 2). Therefore, the basicity of the nitrogen facilitates DPPH radical scavenging, quantified by Equation (2). The positive sign [24] before the 6.72 in the Equation (2) suggests that the model is high specific towards DPPH scavenging.

##### QSAR study on inhibition of lipid peroxidation in linoleic acid emulsion (24 h):

(3) $$\begin{array}{c} {\text{pI}}_{50}=+8.46(\pm 0.47)+2.46(\pm 0.28)\;\text{log}\;P-4.92(\pm 0.36)\;\text{HOMO}\\ +1.96(\pm 0.46)\;4\text{-OH}\;\text{bond}\;\text{length}-6.49(\pm 0.76)\;Q_{\text{S}}\;\\ \text{n}=15;\;\text{r}=0.993;\;\text{s}=0.046;\;\text{F}=103.981;\;{\text{Q}}^{2}=0.991;\;\text{s-PRESS}=0.007 \end{array}$$

Based on the similar behavior of an antioxidant towards DPPH scavenging activity and lipid peroxidation [25,26], which includes dissociation of the O-H bond and release of the hydrogen towards the radical, Equations (2) and (3) characterize the factor of the bond length as important for easy relieving of the hydrogen atom towards the free radical. As the bond is longer, the hydrogen atom is more easily relaxed due to the bond weakening. Furthermore, the ability of the compounds to inhibit peroxidation chain reaction is highly dependent on the coumarin solubility.

##### QSAR study on in vitro hydroxyl radical scavenging activity

(4) $$\begin{array}{l} {\text{pOH}}_{50}=+11.46(0.94)-4.94(0.32)\;\text{HOMO}+7.46(0.24)\;\text{LUMO}+5.96(0.32)\;Q_{0\text{H}}\\ \text{n}=15;\;\text{r}=0.997;\;\text{s}=0.028;\;\text{F}=174.273;\;{\text{Q}}^{2}=0.994;\;\text{s-PRESS}=0.013 \end{array}$$

Free radicals can be easily scavenged on the double bond system. Continued conjugation through the **1**–**10c** coumarin system, consisted of benzene, 4-hydroxy-enol and the C-3 scaffold double bonds (Figure 5) provides the ability for possible hydroxyl radical addition on LUMO coumarin orbital level. The strong participation of LUMO energy and the partial atomic 4-hydroxyl group oxygen charge in the highly specific Equation (4) correlates with high activity of our derivatives.

##### QSAR study on iron chelating ability

(5) $$\begin{array}{c} {\text{pCE}}_{50}=+1.29(0.52)+9.01(0.003)\;\text{CSEV}+0.95(0.33)\;Q_{\text{N}}\\ \text{n}=15;\;\text{r}=0.993;\;\text{s}=0.054;\;\text{F}=43.383;\;{\text{Q}}^{2}=0.894;\;\text{s-PRESS}=0.034 \end{array}$$

In the structure of tested coumarin derivatives there are high voluminous scaffolds which define the compound’s stereochemistry during the chelating of the iron atom. Although the model (Equation 5) does not specify the carbonyl/carboxyl pharmacophores influence, it does specify the great importance of the spatial arrangement of the C-3 residues on the activity. This particular stereochemistry is characterized with the steric molecular descriptor CSEV by Equation (5).

The QSAR statistics are outlined in Table 4 and Table 5. Table 4 presents calculated antioxidant activity values, used for the validation of the models. Excellent validation was further used for the calculation of the activity of the designed compounds (Please see Section 2.7).

Used molecular descriptors were cross-correlated with the experimental activity (Table 5), specifying the part of each descriptor in the related activity.

### 2.7. Structure-Based Design of Novel 4-Hydroxy Coumarin Antioxidants

**Figure 4.**
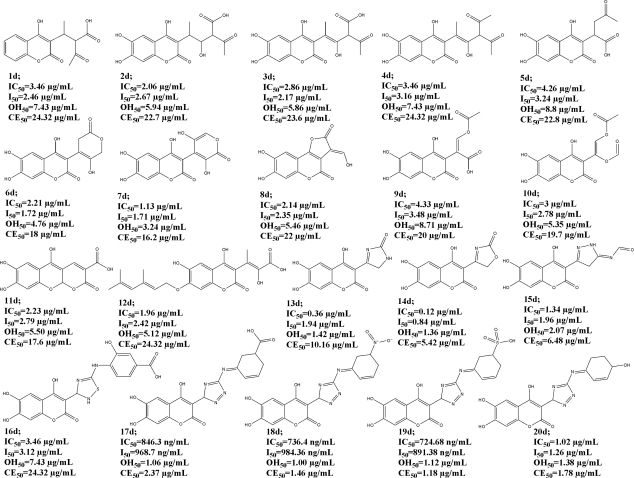
Designed 4-hydroxy-chromene-*2H*-one structures with predicted biological activity *in vitro*.

We have learned how carbonyl/carboxyl and substituted *N*-thiazole moieties contribute to our compounds antioxidant activity and, by the use of QSAR, we designed twenty new improved coumarin structures and calculated their values of potential scavenging and chelating activity (Figure 4).

Therefore, the catehol moiety [11–13] is an important structural part in the design, completing a new backbone with 1,4-pyrone system. The structures were considered in a way that modified C-3 scaffolds, extend the double bond conjugation in many, providing the large number of existing tautomeric forms obtained by the ChemSketch 12 software [27], yet retaining favorable structural properties of the test compounds.

Hence, the number of structural tautomers rises from 3 of compounds **4d** and **8d**, up to even 10 tautomers of **17d**. As the result shows, extended conjugation had improved the overall predicted antioxidant ability. The unfavorable prop-1-ene part of the **b** condensates had been modified by the total removal of the double bond (**1d**, **5d**), addition of one hydrogen group (**12d**) followed by the extension of the carbone chain (**3d**, **4d**), or exploiting carbonyl/carboxyl scaffold which led to various lactone and ether structures (**6**–**11d**). The sulfur atom in the thiazole ring did not contribute to the scavenging activity (Equations (2), (3)), and so is replaced by the construction of oxazole, pyrazole, imidazole and triazole rings (**13**–**20d**). All of the designed compounds had been subjected to the same molecular modeling methods as the original ones and the obtained molecular descriptors were used for the prediction of scavenging and chelating activity using existing QSAR equations. The residue of the triazole ring continued by the Schiff base moiety (**17d**, **18d** and **19d**) is presented as the most successful structural modification, which, along with the appropriate –COOH, –SO_3_H and –NO_2_ ring substituents, reduced the values of coumarin active concentrations to below 1 μg/mL for the IC_50_ and I_50_, and about the same value for the OH_50_ and CE_50_. The most promising structure is, therefore, **19d** with a predicted DPPH scavenging activity of IC_50_ = 724.68 ng/mL. The 3,5-dihydroxy-*2H*-pyran-2-one C-3 structural part (**7d**) contributed the most to the predicted activity of the compounds that ensued from the carbonyl/carboxyl scaffold modifications. By further synthesis and an antioxidant assessment, the design will be vindicated.

## 3. Experimental Section

### 3.1. Chemistry

**Figure 5.**
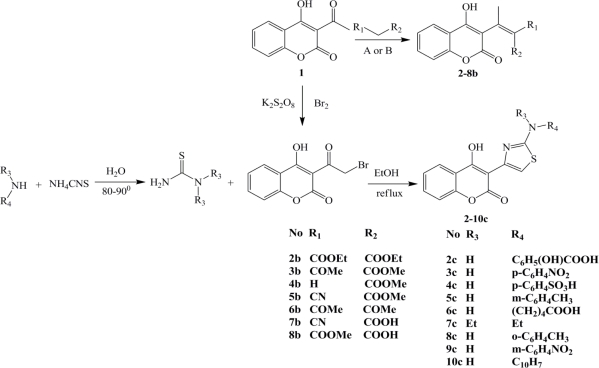
Synthesized coumarin derivatives **1**–**10c**.

In previous papers we had reported synthesis and characterization of the group of 15 substituted chromene-*2H*-one derivatives (**1**–**10c**) (Figure 5) [17,18]. Synthesized compounds were characterized by elemental analysis (C, H, N, O and S) and determination of molecular weights by Mass Spectroscopy (MS). Structural characterization was performed by IR, H^1^ NMR and MS spectra. The purity of synthesized compounds over 98% was confirmed by the HPLC and TLC analysis.

### 3.2. Chemicals

All applied chemicals and reagents were of the highest purity available and purchased from the Sigma-Aldrich Chemical Company (St. Louis, MO, USA), Difco (Sparks, MD, USA) and Merck Laboratory Supplies (Darmstadt, Germany).

### 3.3. The Antioxidant Evaluation *in Vitro*

#### 3.3.1. Determination of Total Antioxidant Capacity by Phosphomolibdenium Assay

The antioxidant activity of the tested compounds was evaluated by the phosphomolibdenium method according to the procedure of Prieto [28]. The assay is based on the reduction of Mo(VI)–Mo(V) by the test compounds and subsequent formation of the green phosphate/Mo(V) complex at acid pH. An aliquot of 100 μL of the methanol solution of the tested compounds (3.901–1000 μg/mL) was combined with 1 mL of reagent solution (0.6 M sulfuric acid, 28 mM sodium phosphate and 4 mM ammonium molybdate). The tubes containing the reaction solutions were incubated at 95 °C for 90 min. Then the absorbance of the solution was measured at 695 nm, using a Perkin-Elmer Lambda 25 UV/Vis spectrophotometer, against blank probe after cooling to room temperature. Methanol (100 μL) in the place of solution of the tested compound was used as the blank. The total antioxidant capacity of the tested samples was calculated according to the Equation (6):

(6) $$\text{TAC}\;(\%)\;=[({\text{A}}_{0}-{\text{A}}_{\text{t}})/{\text{A}}_{0}]\times 100$$

where A_t_ is the absorbance value of the tested sample and A_0_ is the absorbance of the blank sample, in particular time. Ascorbic acid was used as reference standard. The results (TAC) are presented as the μg equivalents of the ascorbic acid per milliliter, obtained from the linear regression analysis. All the experiments were performed in triplicate and the average absorbance was noted for each concentration. As the TAC value is higher, the better is the antioxidant activity. Also, the results of all tests are expressed as EC_50_ values (*i.e.*, TAC_50_, IC_50_, I_50_, OH_50_ or CE_50_) presenting the concentration of the test compound that reduces 50% of the initial free reactive species concentration, calculated as μg/mL, for various concentrations of coumarin derivatives (3.901–1000 μg/mL), with OriginPro 8 statistical software [29] using Nonlinear Curve Fit Growth/Sigmoidal Dose-response function. Percent of inhibition was plotted against concentration, and the equation was the line used to obtain TAC_50_ value. A lower TAC_50_ value indicates greater antioxidant activity.

#### 3.3.2. DPPH Radical Scavenging Assay

The method used by Takao *et al.* [30] was adopted with suitable modifications. DPPH (8 mg) was dissolved in MeOH (100 mL) to obtain a concentration of 80 μg/mL. Serial dilutions (3.901–1000 μg/mL) were carried out with the stock solutions of the compounds **1**–**10c** in methanol. Diluted solutions (2 mL each) were mixed with DPPH (2 mL) and allowed to stand for 30 min and 60 min for any reaction to occur. The absorbance was recorded at 517 nm. Control sample was prepared containing the same volume without test compounds and reference compounds. The radical-scavenging activity of the tested samples, expressed as percentage inhibition of DPPH, was calculated according to the Equation (7).

(7) $$\text{IC}\;(\%)\;=[({\text{A}}_{0}-{\text{A}}_{\text{t}})/{\text{A}}_{0}]\times 100$$

Percent of inhibition after 30 min and 60 min was plotted against concentration, and the equation was the line used to obtain IC_50_ value. A lower IC_50_ value indicates greater antioxidant activity.

#### 3.3.3. Inhibition of Lipid Peroxidation in a Linoleic Acid Emulsion Assay

The ability of coumarin derivatives to inhibit lipid chain peroxidation process was tested in a linoleic acid system [31]. Dilutions (3.901–1000 μg/mL) of the test compounds were prepared in methanol, to add to the linoleic acid emulsion. The linoleic acid emulsion was prepared by mixing 0.2804 g of linoleic acid, 0.2804 g of Tween 20 as emulsifier and 50 mL of phosphate buffer (0.2 M, pH 7) and the mixture was then homogenized. A 0.5 mL of coumarin methanol solution in different concentrations was mixed then in linoleic acid emulsion (2.5 mL, 0.02 M, pH 7) and phosphate buffer (0.2 M, pH 7). The reaction mixture was incubated at 37 °C in the dark to accelerate the peroxidation. Aliquots of 100 μL were taken at different intervals (24–96 h) during incubation. The degree of oxidation was measured by sequentially adding ethanol (4.7 mL, 75%), ammonium thiocyanate sample solution (100 μL, 30%) and FeCl_2_ (100 μL, 0.02 M in 3.5% HCl). After 3 min, the peroxide values were determined by reading the absorbance at 500 nm. Control was performed with linoleic acid but without the tested compounds. Percent inhibition of lipid peroxide generation was calculated using Equation (8).

(8) $$\text{I}\;(\%)\;=[({\text{A}}_{0}-{\text{A}}_{\text{t}})/{\text{A}}_{0}]\times 100$$

Percent inhibition was plotted against concentration, and the equation for the line was used to obtain I_50_. All determinations were carried out in triplicate. The lower I_50_ value indicates greater antioxidant activity.

#### 3.3.4. Hydroxyl Radical Scavenging Activity Assay

The evaluation of coumarins as inhibitors of hydroxyl radical-mediated oxidation was performed with method described by Halliwell *et al.* [32]. The reaction mixture contained 100 μL of tested compounds (with 3.901–1000 μg/mL dilutions) dissolved in ethanol, 500 μL of 5.6 mm 2-deoxy-d-ribose in KH_2_PO_4_/NaOH buffer (50 mM, PH 7.4), 200 μL of premixed FeCl_3_ (104 μM) and EDTA (104 μM) (1:1 v/v) solution, 100 μL of H_2_O_2_ (1.0 mM) and 100 μL of aqueous ascorbic acid (1.0 mM). Tubes were vortexed and heated in water bath at 50 °C for 30 min. Thereafter, 1 mL of 2.8% TCA and 1 mL of 1% TBA were added to each tube with repeat of the 30 min long water heat. The extent of oxidation was estimated from the absorption of solution at 532 nm. The percentage inhibition values were calculated from the Equation (9).

(9) $$\text{OH}\;(\%)\;=[({\text{A}}_{0}-{\text{A}}_{\text{t}})/{\text{A}}_{0}]\times 100$$

Percent inhibition was plotted against concentration, and the equation for the line was used to obtain OH_50_ value. The lower OH_50_ value indicates greater antioxidant activity.

#### 3.3.5. Ferrous Ion Chelating Ability Assay

The ferrous ion chelating activity of methanol coumarin solutions was measured by decrease in absorbance at 562 nm of the iron(II)-ferrozine complex [33]. One milliliter of 0.125 mM FeSO_4_ was added to 1.0 mL of sample (with 1000–3.901 μg/mL dilutions), followed by 1.0 mL of 0.3125 mM ferrozine. The mixture was allowed to equilibrate for 10 min before measuring the absorbance. The ability of the sample to chelate ferrous ion was calculated relative to the control (consisting of iron and ferrozine only) using the Equation (10).

(10) $$\text{CE}\;(\%)\;=[({\text{A}}_{0}-{\text{A}}_{\text{t}})/{\text{A}}_{0}]\times 100$$

Percent inhibition was plotted against concentration, and the equation for the line was used to obtain CE_50_ value. The lower CE_50_ value indicates greater chelating power ability.

### 3.4. Statistical Analysis

All results were expressed as means ±standard deviation (SD). The significance of difference was calculated by one-way ANOVA test and values <0.05 were considered to be significant.

### 3.5. QSAR Study

The QSAR analysis was performed correlating the antioxidant activity presented in Tables 1 and 2, with various molecular descriptors (Table 3) to reveal predictions for the lead optimization in the training set of compounds of synthesized coumarins [17,18]. Although this set is small, it provides QSAR equations that are statistically significant. The results of regression analysis are shown in Equations (1), (2), (3), (4) and (5) and by Figure 3, where *n* is number of molecules, *r* is correlation, *F* is Fisher’s significance factor and *s* is standard deviation. Cross-validation resulted with Q^2^ as the square of predictive power of coefficient and s-PRESS as predictive residual sum of squares. The generation of the QSAR models was taken out with OriginPro 8, by the Multiple Linear Regression (MLR) analysis, using cross validation leave-one-out method. The ratios of observed *vs.* calculated antioxidant activity values and the correlation between the molecular descriptors and the activity are presented in the Tables 4 and 5, respectively.

#### 3.5.1. Molecular Modeling

The initial structures were built in Spartan 2006 for Windows [34] and imported in VegaZZ 2.3.1 [35] molecular modeling package. The Gasteiger charges are assigned with OPLS 2005 force field. Structures have been optimized firstly with AMMP incorporated in VegaZZ 2.3.1. The minimization was performed with 3000 steps Conjugate gradients optimizer model, 0.01 Toler and 0 steepest steps. Further, a 1000 steps Boltzman jump conformation search method was applied searching the flexible torsions only. Temperature had been set to 375.15 K, covering all of the experiment conditions. Torsion root square difference had been set to 60°. Dielectric constant of 33.6 simulated coumarin solution environment, using long range cutoff of 20 and short range of 6 Å. After the cluster analysis, on the selected lowest energy structure full optimization was performed by MOPAC 2009/PM6 [36] Hamiltonian semi empirical method imported in Vega ZZ, by fixing gradient norm as 0.01, with ɛ = 33.6 in order to include solubility of compounds **1**–**10c** in MeOH, according to the experiment conditions. The most stable structures, with determined final heat of formation, were selected as representative conformations in calculation was used for calculation of the electronic descriptors, presented as dominant ones in the antioxidant activity QSAR study of flavonoids, chromones, coumarins and similar plant natural products [21].

Final optimizations were performed by density functional theory (DFT) with the CS Gaussian 03 program [37]. In order to calculate bond dissociation enthalpies of 4-OH groups, optimizations of parent molecules, appropriate radicals and hydrogen radicals were considered. Therefore, parent molecules were optimized by (RO)B3LYP functional and 6-31G(d) basis set, whereas (U)B3LYP/6-31G(d) was applied for radical structures. On the optimized structures full NBO analysis was performed. The geometrical parameters of all stationary points were optimized in methanol solution by CPCM routine. All calculated structures were verified to be local minima (all positive eigenvalues) for ground state structures, by frequency calculation. The zero point vibrational energy and the vibrational contribution to the enthalpy were scaled by a factor of 0.9805 [22]. In order to obtain more reliable relative energy on each stationary point, on the potential energy surface (PES), single-point energy calculations were performed with 6-311G++(2d,2p) diffuse function. The particularly basis set was selected since the optimized molecules are conjugated ones.

#### 3.5.2. Molecular Descriptors

Molecular descriptors generated for this study were electronic and steric. Solubility factor was considered for the lipid peroxidation environment. Electronic molecular descriptors obtained after optimization were: HOMO and LUMO energies, Δ*E* HOMO-LUMO calculated as the difference of the energies, dipole, total energy, electronic energy, O–H bond length, with Mulliken population analysis applied for the calculation of the partial atomic charges (*Q*). The molecular descriptors values are presented in the Table 3.

During the DPPH and lipid peroxidation assays, the hydroxyl group proton from the antioxidant is transferred towards the free radical. The process is, taking peroxyl radical (ROO.) as a sample, presented by Equation (11) [22].

(11) ROO+ArOH→ROOH+ArO

Covering the fact that process is governed by the O-H bond dissociation enthalpy (BDE) of the antioxidant, we calculated BDE *via* the formula: BDEs = *H*_KOH_ − *H*_KO_ − *H*_H_, where *H*_KOH_ is the enthalpy for the parent coumarin molecule, *H*_KO_ is the enthalpy for radical generated after H-abstraction and *H*_H_ is the enthalpy for hydrogen radical.

Coumarin antioxidant potential is increased with the extended conjugation, including bond orders [21] and some steric parametes like Connolly Accessible Area (CAA), Connolly Molecular Area (CMA), Connolly Solvent-Excluded Volume (CSEV) and principal moment of inertia, calculated by VEGA ZZ, as useful molecular descriptors.

## 4. Conclusions

Within the series of the examined coumarin derivatives in accordance with the presented experimental results for the total antioxidant capacity and the affinity towards DPPH, lipid peroxide and hydroxyl radicals, it was concluded that compounds **2b**, **6b**, **2c** and **4c** are notable antioxidants. Therefore, these compounds could be practically applied as antioxidant agents and as the starting compounds for future selective modifications of the coumarin molecule according to performed SAR, QSAR and experimental conditions based theoretical studies on their activity.

## Figures and Tables

**Table 1.** Total antioxidant capacity and DPPH radical scavenging activity of synthesized coumarin derivatives *in vitro*.

**Comp.**	**^a^TAC (μg/mL)**	**^b^TAC_50_ (μg/mL)**	**^c^IC_50_ (μg/mL)**	
			**30 min**	**60 min**
**1**	121.46 ± 0.28^b^	97.45 ± 0.31^d^	133.70 ± 0.24	87.47 ± 0.24
**2b**	278.24 ± 0.36	47.65 ± 0.24	6.2 ± 0.11	4.6 ± 0.26
**3b**	54.08 ± 0.76	197.62 ± 0.21	44.93 ± 0.16	8.80 ± 0.14
**4b**	138.32 ± 0.87	84.57 ± 0.65	41.64 ± 0.14	9.93 ± 0.22
**6b**	212.12 ± 0.26	50.59 ± 0.12	5.14 ± 0.06	2.45 ± 0.17
**7b**	324.01 ± 0.28	35.69 ± 0.17	246.63 ± 0.31	135.01 ± 0.31
**8b**	106.64 ± 0.15	99.71 ± 0.28	37.76 ± 0.21	11.28 ± 0.19
**2c**	514.24 ± 0.64	33.35 ± 0.24	4.94 ± 0.08	6.97 ± 0.25
**3c**	86.14 ± 0.95	132.66 ± 0.16	29.07 ± 0.04	9.22 ± 0.17
**4c**	742.67 ± 0.28	17.25 ± 0.15	4.72 ± 0.03	3.54 ± 0.32
**5c**	198.84 ±0.24	53.16 ± 0.09	68.56 ± 0.07	66.54 ± 0.26
**6c**	164.61 ± 0.32	72.35 ± 0.15	115.42 ± 0.15	94.30 ± 0.24
**7c**	224.26 ± 0.31	46.67 ± 0.11	161.73 ± 0.46	93.58 ± 0.17
**8c**	219.94 ± 0.56	47.18 ± 0.28	140.48 ± 0.26	60.31 ± 0.06
**9c**	82.22 ± 0.96	136.94 ± 0.53	13.72 ± 0.25	4.79 ± 0.03
**10c**	26.76 ± 0.48	219.43 ± 0.89	78.25 ± 0.11	76.41 ± 0.05
**Asc**	/	/	24.17 ± 0.07	15.61 ± 0.04
**BHT**	/	/	8.62 ± 0.02	6.05 ± 0.01

a Total antioxidant capacity of the coumarin derivatives expressed as μg equivalents of ascorbic acid per milliliter;

b Total antioxidant capacity of the coumarin derivatives; TAC_50_, the concentration of coumarin required to inhibit 50% of Mo(VI) reduction;

c DPPH radical scavenging activity;

d Results are mean values ± SD from at least three experiments.

**Table 2.** Lipid peroxide, hydroxyl radical scavenging and chelating effect of coumarin derivatives *in vitro*.

**Comp.**	**^a^I_50_ (μg/mL)**			**^b^OH_50_ (μg/mL)**	**^c^CE_50_ (μg/mL)**
	**24 h**	**48 h**	**72 h**		
**1**	26.31 ± 0.31	55.23 ± 0.22	55.23 ± 0.32	17.77 ± 0.15	475.24 ± 0.21
**2b**	7.77 ± 0.12	16.85 ± 0.15	13.01 ± 0.14	17.19 ± 0.06	45.0 ± 0.54
**3b**	12.08 ± 0.12	28.07 ± 0.41	10.46 ± 0.28	32.21 ± 0.41	57.35 ± 0.34
**4b**	18.96 ± 0.04	37.89 ± 0.28	80.95 ± 0.24	84.04 ± 0.02	62.5 ± 0.11
**6b**	<3.901	10.13 ± 0.16	10.76 ± 0.16	14.32 ± 0.15	28.64 ± 0.28
**7b**	76.17 ± 0.25	216.85 ± 0.22	167.66 ± 0.46	9.89 ± 0.03	60.74 ± 0.41
**8b**	11.49 ± 0.24	18.07 ± 0.31	16.02 ± 0.24	37.82 ± 0.08	55.18 ± 0.13
**2c**	6.72 ± 0.37	17.89 ± 0.34	7.07 ± 0.34	9.81 ± 0.03	34.64 ± 0.11
**3c**	24.94 ± 0.34	12.02 ± 0.09	51.53 ± 0.13	19.83 ± 0.24	5.5 ± 0.08
**4c**	<3.901	26.31 ± 0.16	10.09 ± 0.27	5.94 ± 0.04	43.19 ± 0.02
**5c**	24.49 ± 0.37	100.94 ± 0.17	51.33 ± 0.27	70.51 ± 0.63	52.04 ± 0.45
**6c**	33.46 ± 0.48	28.66 ± 0.17	83.29 ± 0.19	30.32 ± 0.34	62.25 ± 0.27
**7c**	53.13 ± 0.19	40.28 ± 0.36	119.34 ± 0.24	36.88 ± 0.72	54.77 ± 0.16
**8c**	33.01 ± 0.16	731.60 ± 0.28	80.98 ± 0.31	69.76 ±0.28	52.81 ± 0.25
**9c**	5.88 ± 0.34	10.06 ± 0.34	12.23 ± 0.17	24.32 ± 0.11	7.76 ± 0.05
**10c**	28.20 ± 0.26	62.34 ± 0.19	57.06 ± 0.29	54.82 ± 0.03	51.64 ± 0.26
**Asc**	246.14 ± 0.3	514.36 ± 0.16	>1000	160.55 ± 0.19	76.31 ± 0.25
**BHT**	<7.81	<7.81	<7.81	33.92 ± 0.34	85.48 ± 0.17

a Lipid peroxidation scavenging capacity of coumarin derivatives;

b Hydroxy radical scavenging capacity of coumarin derivatives;

c Chelating capacity of coumarin derivatives.

**Table 3.** Bond dissociation enthalpies and the relevant molecular descriptors for the QSAR studies.

**Comp.**	**E1**	**D1**	**D2**	**D3**	**D4**	**D5**	**D6**	**D7**	**D8**	**D9**
**1**	114.32	−9.99	−1.49	−8.50	0.99	−0.62	0	0	124.41	−0.529
**2b**	199.74	−10.01	−1.56	−8.45	0.99	−0.66	0	0	203.33	−1.561
**3b**	227.25	−9.96	−1.54	−8.42	1.04	−0.63	0	0	165.18	−1.318
**4b**	224.96	−9.98	−1.45	−8.53	1.02	−0.61	0	0	192.32	−0.035
**6b**	205.84	−10.04	−1.65	−8.39	1.03	−0.67	0	0	171.66	−1.679
**7b**	211.94	−10.04	−1.69	−8.35	1.06	−0.69	0	0	173.41	−1.765
**8b**	216.27	−9.04	−1.45	−7.59	0.96	−0.66	0	0	246.34	−1.709
**2c**	242.37	−9.00	−1.95	−7.05	1.55	−0.61	−0.59	0.25	233.72	1.216
**3c**	255.69	−8.99	−1.62	−7.37	1.53	−0.46	−0.62	0.27	249.55	3.129
**4c**	239.26	−8.90	−1.13	−7.77	1.63	−0.65	−0.31	0.29	225.94	0.702
**5c**	244.56	−8.92	−0.94	−7.78	1.36	−0.70	−0.66	0.47	230.13	2.904
**6c**	249.78	−8.89	−0.95	−7.94	1.59	−0.67	−0.68	0.27	208.37	1.921
**7c**	269.29	−8.91	−1.07	−7.84	1.69	−0.63	−0.69	0.26	225.74	1.856
**8c**	251.48	−8.97	−1.76	−7.21	1.61	−0.68	−0.62	0.24	237.20	3.380
**9c**	281.35	−8.87	−1.23	−7.64	0.99	−0.65	−0.64	0.25	248.00	3.129
**10c**	271.52	−8.79	−1.16	−7.63	0.99	−0.65	−0.65	0.26	222.34	3.603

a E-energy: E1 *BDEs* (kcal/mol);

b D-descriptor: D1: *HOMO* (eV); D2: *LUMO* (eV); D3: *H-L gap* (eV); D4: *4-OH bond length* (Å); D5: *Q_0H_*; D6: *Q_N_*; D7: *Q_S_*; D8: *CSEV* (Å^2^); D9: log *P*.

**Table 4.** Observed *vs.* calculated values of coumarin derivatives antioxidant activity from QSAR studies.

**Comp.**	**pTAC_50_**		**pIC_50_**		**pI_50_**		**pOH_50_**		**pCE_50_**	
	**Obser.**	**Calc.**	**Obser.**	**Calc.**	**Obser.**	**Calc.**	**Obser.**	**Calc.**	**Obser.**	**Calc.**
**1**	4.01	3.89	4.06	4.26	4.57	4.78	4.71	4.80	3.32	3.42
**2b**	4.32	4.71	5.34	5.43	5.12	5.18	4.76	4.72	4.34	4.37
**3b**	3.70	3.69	5.06	5.11	4.92	5.14	4.49	4.62	4.24	4.36
**4b**	4.07	4.88	5.00	5.09	4.72	4.82	4.06	4.13	4.20	4.44
**6b**	4.29	4.28	5.61	5.65	5.41	5.41	4.84	5.02	4.54	4.44
**7b**	4.45	4.76	3.87	3.75	4.12	4.18	5.00	5.13	4.22	4.52
**8b**	4.00	3.89	4.95	4.96	4.94	5.08	4.42	4.47	4.26	4.36
**2c**	4.47	4.91	5.16	5.25	5.17	5.48	5.00	5.23	4.46	4.52
**3c**	3.88	3.92	5.03	5.13	4.60	4.92	4.70	4.86	5.26	5.31
**4c**	4.76	5.01	5.45	5.69	5.41	5.61	5.23	5.55	4.36	4.26
**5c**	4.27	4.14	4.12	4.06	4.66	4.63	4.15	4.26	3.28	3.34
**6c**	4.14	4.08	4.63	4.80	4.47	4.73	4.52	4.72	4.21	4.21
**7c**	4.33	4.36	4.22	4.42	4.27	4.38	4.43	4.44	4.26	4.26
**8c**	4.32	4.445	5.32	5.68	4.48	4.56	4.16	4.22	4.28	4.45
**9c**	3.86	3.15	4.12	4.12	5.23	5.53	4.61	4.71	5.11	5.34
**10c**	3.66	3.49	4.06	4.26	4.55	4.72	4.12	4.32	4.29	4.26

**Table 5.** Correlation matrix of molecular descriptors values and antioxidant activity.

	**D1^a^**	**D2**	**D3**	**D4**	**D5**	**D6**	**D7**	**D8**	**D9**	**A1^b^**	**A2**	**A3**	**A4**	**A5**
**D1**	1.00													
**D2**	0.48	1.00												
**D3**	0.96	0.91	1.00											
**D4**	0.48	0.51	0.37	1.00										
**D5**	0.36	0.29	0.91	0.96	1.00									
**D6**	0.23	0.79	0.61	0.29	0.51	1.00								
**D7**	0.22	0.24	0.41	0.34	0.55	0.84	1.00							
**D8**	0.11	0.13	0.24	0.09	0.38	0.31	0.41	1.00						
**D9**	0.26	0.42	0.35	0.14	0.18	0.31	0.29	0.19	1.00					
**A1**	0.97	0.74	0.00	0.00	0.87	0.64	0.79	0.00	0.12	1.00				
**A2**	0.00	0.00	0.00	0.98	0.00	0.78	0.98	0.00	0.26	0.00	1.00			
**A3**	0.64	0.00	0.00	0.93	0.00	0.00	0.68	0.00	0.94	0.00	0.68	1.00		
**A4**	0.76	0.95	0.00	0.00	0.98	0.00	0.00	0.00	0.09	0.00	0.34	0.17	1.00	
**A5**	0.00	0.00	0.00	0.00	0.00	0.91	0.00	0.98	0.14	0.00	0.00	0.00	0.00	1.00

a D- Please see Table 3;

b A1: *pTAC_50_*; A2: *pIC_50_*; A3: *pI_50_*; A4: *pOH_50_*; A5: *pCE_50_*.

## Abbreviations:

NBO: Natural Bond Orbital;
OPLS: Optimized Potentials for Liquid Simulations;
AMMP: Another Molecular Mechanics Program;
PM6: Parametric Method 6;
B3LYP: Becke, three-parameter, Lee-Yang-Parr.
